# An ontology-based nurse call management system (oNCS) with probabilistic priority assessment

**DOI:** 10.1186/1472-6963-11-26

**Published:** 2011-02-04

**Authors:** Femke Ongenae, Dries Myny, Tom Dhaene, Tom Defloor, Dirk Van Goubergen, Piet Verhoeve, Johan Decruyenaere, Filip De Turck

**Affiliations:** 1 Ghent University - IBBT, Department of Information Technology (INTEC), Gaston Crommenlaan 8, bus 201, 9050 Ghent, Belgium; 2 Ghent University Hospital, Nursing department, De Pintelaan 185, 9000 Ghent, Belgium; 3 Ghent University, Faculty of Medicine and Health Sciences, Nursing Science, Ghent, Belgium, Pintelaan 185, 9000 Ghent, Belgium; 4 Ghent University, Department of Industrial Management, Technology Park 903, 9052 Zwijnaarde, Belgium; 5 Televic R&D, Leo Bekaertlaan 1, 8870 Izegem, Belgium; 6 Ghent University Hospital, Intensive Care Department, De Pintelaan 185, 9000 Ghent, Belgium

## Abstract

**Background:**

The current, place-oriented nurse call systems are very static. A patient can only make calls with a button which is fixed to a wall of a room. Moreover, the system does not take into account various factors specific to a situation. In the future, there will be an evolution to a mobile button for each patient so that they can walk around freely and still make calls. The system would become person-oriented and the available context information should be taken into account to assign the correct nurse to a call.

The aim of this research is (1) the design of a software platform that supports the transition to mobile and wireless nurse call buttons in hospitals and residential care and (2) the design of a sophisticated nurse call algorithm. This algorithm dynamically adapts to the situation at hand by taking the profile information of staff members and patients into account. Additionally, the priority of a call probabilistically depends on the risk factors, assigned to a patient.

**Methods:**

The *ontology-based Nurse Call System (oNCS) *was developed as an extension of a *Context-Aware Service Platform*. An ontology is used to manage the profile information. Rules implement the novel nurse call algorithm that takes all this information into account. Probabilistic reasoning algorithms are designed to determine the priority of a call based on the risk factors of the patient.

**Results:**

The *oNCS *system is evaluated through a prototype implementation and simulations, based on a detailed dataset obtained from Ghent University Hospital. The arrival times of nurses at the location of a call, the workload distribution of calls amongst nurses and the assignment of priorities to calls are compared for the *oNCS system *and the current, place-oriented nurse call system. Additionally, the performance of the system is discussed.

**Conclusions:**

The execution time of the nurse call algorithm is on average 50.333 ms. Moreover, the *oNCS system *significantly improves the assignment of nurses to calls. Calls generally have a nurse present faster and the workload-distribution amongst the nurses improves.

## Background

### Introduction

Information technology is widely adopted in modern medical practice, especially to support administrative tasks, electronic patient records (EPRs) and data management [1,2]. The challenge today is that several data sources and devices have to be manually combined and consulted by the staff members to take advantage of this information, even when carrying out one single task. This is a time consuming job [3]. An underdeveloped area of solution for this problem is the use of context-aware techniques to automatically exploit the medical information available to improve continuous care and personalize healthcare. This implies an emerging demand for the integration and exploitation of the heterogeneous information available from all the wireless devices, patient records and medical data. Building context-aware applications on top of an ontology can ideally do this. An important way to coordinate work, communicate and provide continuous care is by making use of a nurse call system.

**Figure 1:**
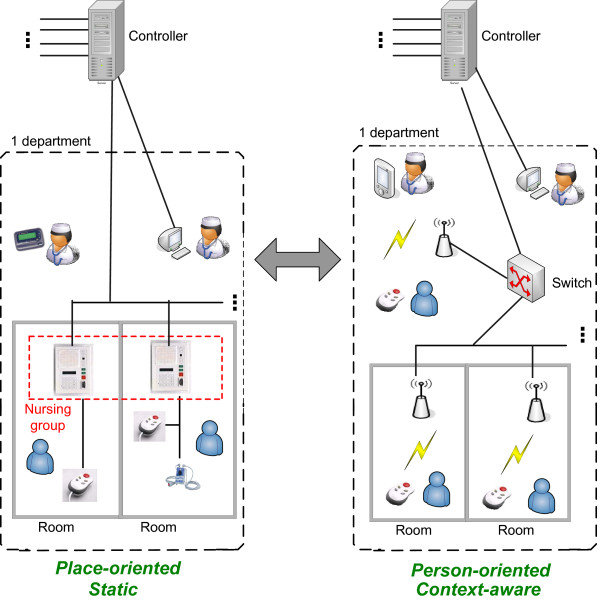
**The traditional place-oriented and static nurse call system vs. the future person-oriented and context-aware approach**. The architecture of traditional nurse call systems can be viewed in the left part of the figure. Each room has at least one button which can be used by the patient to call a nurse. All the buttons in a room are connected to a *Node*. All the *Nodes *of a department are connected with each other and a *Controller*. The *Controller *has the intelligence to know what must happen when a call is made, for example which nurses must be called. A PC can be used to configure the controller. The nurses possess beepers or portable phone on which they can receive calls. Within a department, the *Nodes *can be further divided into different, possibly overlapping, nursing groups. A nurse will only receive calls of the nursing groups that this nurse is assigned to. The proposed architecture of the person-oriented and context-aware nurse call system can be viewed in the right part of the figure. Each patient has a mobile button so that they can walk around freely and still make calls. These calls are picked up by the sensor network and processed by the *Controller*. The *Controller *calls a nurse to handle the call. The nurse receives the call on his or her PDA.

The architecture of traditional place-oriented nurse call systems can be viewed in the left part of Figure 1. Each room has at least one button which can be used by the patient to call a nurse. All the buttons in a room are connected to a *Node*. All the *Nodes *of a department are connected with each other and a *Controller*. The *Controllers *are the heart of the system. They contain the intelligence to know what must happen when a call is made, for example which nurses must be called.

The *Nodes *can be divided into different departments which each have their own specific settings. Within a department, the *Nodes *can be further divided into different, possibly overlapping, nursing groups. Each group can have his own configuration settings concerning for example the priorities of the different kinds of calls. Each nurse, who is identified by his or her beeper or portable phone number inside the system, is assigned to at least one nursing group. A nurse will only receive calls of the nursing groups that this nurse is assigned to. The advantage of using nursing groups is that patients who need more attention can be equally divided amongst the groups or be put in a separate group to distribute the workload better amongst the nurses.

The traditional nurse call algorithm consists of predefined links of beeper or portable phone numbers to rooms. To make a call the patient pushes one of the fixed buttons in his room. All the beepers and portable phones of the nurses, who are in the nursing group that this room belongs to, are activated. The nurses decide on their own if they are going to interrupt their current task to answer the call or not. The nurse who reaches the room first will handle the call.

On one hand, the current nurse call systems are place-oriented. When a patient makes a call with a button that is fixed to a wall of a room, the called nurse simply goes to the room where the call came from. Herewith two important assumptions are made: the patient must still be in the room and it must be the patient who lies in the room that made the call. A patient can also only make calls inside his room. It is dangerous to become unwell, e.g. heavy respiratory or heart problems, inside a hallway, staircase or outside. This leads to patients being confined to their room to ensure their safety.

On the other hand, the system does not take into account various factors specific to a situation, such as the risk factors of a patient or the characteristics of the staff. Multiple nurses, namely all the nurses inside the nursing group that this room belongs to, are called. They have to decide for themselves if they are going to interrupt their current task to answer the call. They have no information about the priority and the kind of call or about the patient to guide them in this decision. If they interrupt their current work, which can also be a call, they have to remember themselves that they have to return to it. It is possible that more than one nurse goes to answer the call. This makes the whole system somewhat unreliable and inefficient.

Overall there is a transition to a world with more mobile and wireless devices [4]. In a study of Miller [5] the user friendliness and influence on nursing time is compared of two nurse call systems. The first system is comparable to the nurse call system detailed above. In the second system the staff members additionally were given locator badges through which they could be constantly tracked. 80% of the participants in the study preferred the second system to the first one. This is because a lot of time in a hospital is spent on *trying to find someone*. This claim is supported by a study of Linden [6] which found that almost 10% of nursing time is spent *looking for someone*. By using the locator badges this became an easier and less time-consuming task. Thus in the future, there will be an evolution to a mobile button for each patient so that they can walk around freely and still make calls, as can be seen in the right part of Figure 1.

This evolution implies a lot of changes, for example the nurse has to go to the exact location of the patient and the patients can make calls from anywhere in or outside the hospital. This huge impact is comparable to the introduction of the mobile phone. In the past we used to call to a telephone (a place) and ask for the correct person. Now we call a mobile phone en we immediately expect to have the right person on the line.

Context information becomes increasingly important in a world with more and more wireless devices that have to be in touch with the environment around them. Lots of problems in current nurse call system are caused by the fact that they do not take the context information into account. The study of Linden also found that nurses are often called for tasks that could also be done by a less qualified staff member. Another study by Miller [7] supports this claim by concluding that on average 51% of the time registered nurses perform activities outside their role definition and do not require their level of knowledge and ability. Rerouting these kinds of calls to other staff members might greatly improve response time and patients satisfaction. Studies of call light use have also found that a large amount of calls are accidental calls [8]. Finding a way to indicate these calls might greatly improve the work pressure put on nurses and caregivers. Some features of the nurse call system which were identified as favorable to the performance of the staff are: locating staff, direct room-to-room communication and identification of the importance of calls, e.g. accidental or not or specifying condition and history of the patient.

In this article a novel software platform, the *ontology-based Nurse Call System *(*oNCS*), is proposed that supports the transition to mobile and wireless nurse call buttons. Additionally, this platform efficiently manages the profiles of the staff members and the patients by encoding this context information into an ontology [9]. A new nurse call algorithm was developed that dynamically adapts to the situation at hand by taking the profile information into account such as the location and the characteristics of the staff and the patients, the current tasks of the staff members and the priorities of the calls. All this information is used to find the best staff member to handle a specific call and thus eliminate the above mentioned problems currently present in nurse call systems.

To clearly illustrate the person-oriented nature of the platform, the context information about the risk factors of a patient is used to dynamically determine the priority of the call this patient is making. By using probabilistic reasoning algorithms, the probability that a specific call made by a specific patient has a certain priority can be determined. These probabilities are derived from the different risk factors this patients has because they will influence the probability that a patient makes urgent calls. All these probabilistic values are combined in an intelligent manner to determine the most suitable priority for this call.

### Objectives

The aim of this research is the design of a software platform that enables the transition to mobile and wireless nurse call buttons in hospitals and nursing homes and employs an intelligent nurse call algorithm that takes the profiles of the staff members and patient into account. The platform should offer the advanced features listed below:

• *Profile management:*In order to achieve a nurse call algorithm that adapts to the situation at hand, context information about the profiles of patients and staff members should be managed efficiently.

• *Dynamic priority assessment:*Instead of statically defining the priority of a call in advance, it should depend on the profile of the patient and more specifically on his or her risk factors. As patients with a certain profile can still make calls of varying priority, this information should be modeled probabilistically. As it is difficult to accurately determine the exact probability with which a patient with a certain profile will make a call of a certain priority, the platform should be able to handle probabilistic intervals.

• *Mobile:*The platform should give the patients enough mobility. They should be able to wander around the whole hospital and a limited area outside of the hospital for example the smoking area and the parking lot. They should be able to make calls in all these areas without their call getting lost because of bad reception. The mobile buttons should also be easy to operate.

• *Location-Aware:*The platform should be able to detect the locations of patients and staff members in a sufficiently accurate way and take this information into account when finding a suitable staff member to handle a call. This data should be constantly monitored and transparently delivered to the system.

• *Efficient staff assignment:*The nurse call algorithm should ensure that an optimal matching is achieved between the profiles of the staff members and the profile of the patient, when finding a suitable staff member to handle a call. An efficient workload distribution should be achieved between all the staff members who can handle each type of calls. A good balance between safety and cost should be achieved. The quality of care may not be undermined.

• *Reliable:*Four kinds of faults can occur: the server can go down, a call is not delivered to the server, a call is not delivered to the PDA of the staff member or the location information cannot be received or is inaccurate. The platform has to be able to cope with each of these situations. Calls may never be lost and it should always be able to call at least one staff member. A good logging infrastructure is needed to ensure that it is always known which patients made calls, which staff members handled them and how long it took until a staff member was at the location.

• *Performance:*The performance of the platform and the algorithms should be such that general guidelines can be imposed, for example, the guideline that stipulates that at least one staff member should arrive at the location of the patient within 3 minutes when an urgency call was made and within 5 minutes for other calls. As these time constrictions include walking to the patient, the time needed by the algorithm to assign a suitable staff member to a call should be negligible.

• *Generic:*It should be possible to plug-in new components, independent of implementation languages, operating systems and hardware by providing generic interfaces. New applications to visualize and input information from and into the platform should be easy to develop and plugged into the system.

• *Scalability:*The platform should be able to handle to large amount of profile information that is available about all the staff members and patients currently in the hospital. It should also be able to handle the large amount of calls that can daily enter the system.

### Related Work

On one hand, general purpose frameworks and models have been proposed that capture general concepts about contexts in an ontology and provide reasoning on this contextual model. For example, In Preuveneers et al. [10] an adaptable and extensible ontology is proposed for creating context-aware computing infrastructures, ranging from small embedded devices to high-end service platforms. In Gu et al. [11] an OSGi-based infrastructure for context-aware applications is proposed and Chen et al. [12] defined a context ontology based on OWL to support ubiquitous agents. However, all these frameworks are not specific for the healthcare domain.

On the other hand, many ontologies have been developed for the healthcare domain to model context, mainly for medical decision making [13,14]. However, some ontologies that address the continuous care context have also been developed. For example, the ontology *OntHos *[15] was developed to model hospital scenarios and to facilitate their interoperability and Kataria et al. [16] implemented an ontology for an intelligent hospital ward to address data sharing and semantic heterogeneity. However, these papers do not address the context-aware reasoning that should take place on top of the ontology.

Yao et al. [17] tried to fill the gap between general purpose context-aware frameworks and a healthcare domain specific ontology. They propose the *CIHO *model, an extensible hospital ontology to represent, manipulate and access hospital information in intelligent environments. Additionally, they present examples of ontology reasoning and rule-based reasoning to show how context-aware services can be built. However, no complete service was built and evaluated.

In this paper we build further on the work of Yao et al. to unite the research on ontologies for continuous care with the research on frameworks for context-aware applications. A general purpose context-aware framework, namely the *Context-Aware Service Platform *(*CASP*) [18], is extended with a continuous care ontology which models the profile information of staff members and patients and context information about tasks and nurse calls. The main contribution of our work is the incorporation of probabilistic information in the ontology and the development of sophisticated probabilistic reasoning algorithms to achieve a sophisticated context-aware application. Additionally, the novel nurse call system was thoroughly evaluated through simulations based on realistic data.

### Paper organization

The remainder of this paper is organized as follows. The *Methods *Section starts with a general description of the platform. Secondly, it is detailed how the profiles of the staff members and patients are managed by employing an ontology. It is also explained how information about the priorities of calls can be modeled so that it depends probabilistically on the risk factors of patients. Thirdly, the developed algorithms are presented. It is detailed how the probabilistic information can be used to determine the priority of a call. An overview of the novel nurse call algorithm is given that takes all the profile information in the ontology into account to find the best staff member to handle a call. The fourth subsection describes the implementation details. To test and demonstrate the advantages and performance of the system, a simulation was set up with realistic data provided by Ghent University Hospital [19]. The set-up is detailed in the final subsection of the *Methods *Section, while the results are discussed in the *Results *Section. The *Discussion *Section presents a critical discussion of the platform and its benefits. Finally, the main conclusions are highlighted in the last Section.

## Methods

### General concept

**Figure 2:**
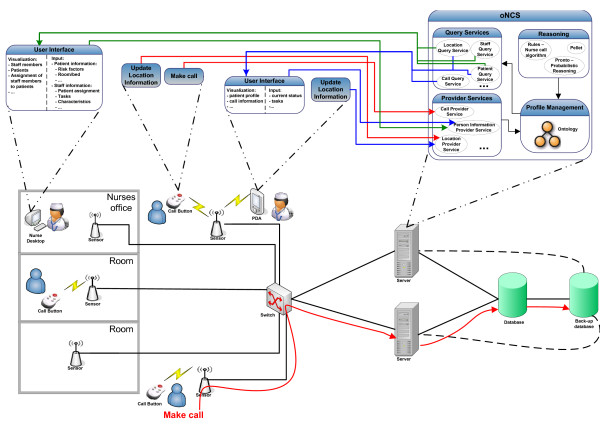
General concept of the *oNCS platform *with probabilistic risk assessment and profile management

The main functionality of the person-oriented nurse call system with probabilistic risk assessment is to provide efficient support for wireless nurse call buttons and to employ a more sophisticated nurse call algorithm that takes the profiles of the staff members and patients into account. The general concept of the platform is illustrated in Figure 2.

Patients can walk around freely in the hospital with their wireless nurse call buttons. These buttons periodically broadcast a message which is picked up by the nearby sensors. The large number of available sensors guarantees that another sensor can pick up the message in case the closest one is malfunctioning. This information then travels through the switch to the back-end server, as can be seen in the bottom part of Figure 2. Existing state-of-the-art algorithms [20,21] can be used to detect the accurate location of the patient out of this information by taking, for example, the signal strength perceived by the various sensors into account. When the location cannot be calculated or is inaccurate, the previous location information is used until the next broadcast is detected. When the patient makes a call, a call message is sent in a similar manner. In this case the server does not only update the location of the patient, but also initiates the algorithm to find the most appropriate staff member to handle the call. The location of the patient is updated and monitored until a staff member is at the scene to handle the call.

Each staff member has a PDA which provides a user-friendly *Graphical User Interface *(*GUI*). Information about the patients such as their risk factors or location can be requested. The PDA also notifies the staff member of calls that this staff member has been assigned to. The staff member is able to request information about the call such as where it originated from and what the priority is. The staff member can also indicate if he/she is going to handle the call or not. The sensor network is used to automatically detect that the staff member is at the location of the patient and is thus handling the call.

A desktop is available in each department which provides the head nurse with a *GUI *to input and visualize information about the department. The head nurse can input information about the patients, such as their risk factors or which rooms they occupy, and about the staff members, such as their characteristics or the patients they are responsible for. Information about the department is displayed in an overview window which shows which nurse has been assigned to which patient and where all the staff members and patients currently are. By clicking on a staff member or patient, the head nurse can view additional information about this person.

The new *ontology-based Nurse Call System *(*oNCS*) *platform *handles all the communication to and from these devices. The platform contains an ontology which is used to model all the profile information about the patients and staff members. The platform offers a wide range of *Web Service *[22] methods to transparently gain access to this information. Transparent access means that applications or users, who want to input data into the *oNCS system *or extract data from it, do not have to be aware of the underlying structure of the data e.g. the ontology or database. The *Web Service *provides an interface to input or extract data from the system, while the translation to the correct ontology or database query is kept completely hidden. This *Web Service *can be called from anywhere in the network. The *Provider Services *transform the inputted information to data that can be inserted in the ontology. The *Query Services *transform the data from the ontology to information that can be processed by the applications on the PDAs or desktops. These generic *Web Services *make it easy to write and plug new applications into the platform. This is further detailed in *The oNCS platform *Subsection of the *Implementation details *Section.

The ontology contains all the necessary context information about the hospital such as information about the profiles of the staff members, the profiles of the patients and the calls. It also contains information about the risk factors of the patients. General information about the priorities of calls is modeled with probabilistic intervals in the ontology. These priorities thus depend probabilistically on the risk factors of the patients. The ontology is further detailed in the *Profile Management *Section.

*Rules *implement the novel nurse call algorithm that takes all the information in the ontology into account to find the best staff member to handle a call. The matching of a staff member to a call is not solely based on the fact that this staff member is responsible for the patient. Additional information such as the location of the staff members and the patient, the priority of the call, the characteristics of the staff member and the patients and the current task of the staff member are taken into account. The *Rules *are automatically triggered when a new call is inserted into the ontology. As a result the call is send to the PDA of the staff member who has been chosen to handle it. To ensure the reliability of the system, the algorithm also contains a time-out procedure. When a staff member has not indicated that he/she is going to handle the call within a certain amount of time, the call is launched again. The algorithm is further explained in *The nurse call algorithm *Subsection of the *Algorithms *Section.

The priority of a call is determined by reasoning algorithms that reason on the probabilistic information in the ontology about the risk factors of a patient. This priority can then be taken into account in the nurse call algorithm. The probabilistic reasoning algorithms are detailed in the *Priority Assessment of a call *Subsection of the *Algorithms *Section.

### Profile management

In order to achieve a nurse call algorithm that adapts to the situation at hand, context information about the profiles of patients and staff members should be managed efficiently. Ontologies can be used to structure and represent knowledge about a domain in a formal way [9]. This knowledge can then easily be shared and reused. Because of the foundation of ontologies in *First-Order Logic *(*FOL*), the models and description of the data in these models can be formally proofed. It can also be used to detect inconsistencies in the model as well as infer new information out of the correlation of this data. This proofing and classification process is referred to as reasoning.

To develop the *oNCS ontology*, a couple of concrete situations were studied in cooperation with the experts in the domain of nurse call systems at Televic NV [23]. For each situation the relevant context information was extracted and the ontology was augmented with it. It took several iterations and meetings with domain experts to get the desired ontology [24,25]. The subsections below highlight the most important parts of the ontology.

#### Profile model of the staff members and patients

**Figure 3:**
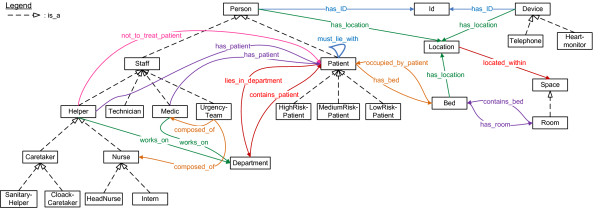
**Fragment of the ontology that models the context information about the staff members and patients**. Fragment of the ontology which models the patients and staff members of the hospital who can answer calls. The squares represent the classes. The arrows with the striped lines indicate subclass relationships. The other arrows and lines indicate relations between classes (object properties).

First, the patients and the staff members of the hospital who can answer calls were modeled, as can be seen in Figure 3. The current location is tracked for each staff member and patient. All staff members have associated beepers and/or portable phone numbers. It is also modeled on which departments a staff member works and on which department a patient lies. Some information is also maintained for administrative purposes such as names, IDs, beds and rooms.

Helpers can have different specializations. Two special types of nurses, namely head nurses and interns, and caretakers have been defined. Sanitary helpers are responsible for *caring *tasks such as cleaning a bed or fluffing a pillow. Family caregivers are volunteers.

In the place-oriented system, each helper was associated with a nursing group. However, in the person-oriented system it is more logical to associate each helper with a group of patients for whom this helper is responsible. This makes the system very flexible, as these groups can be dynamically adapted to equally divide the work load among the different helpers. Each medical staff member is also responsible for one or more patients.

**Figure 4:**
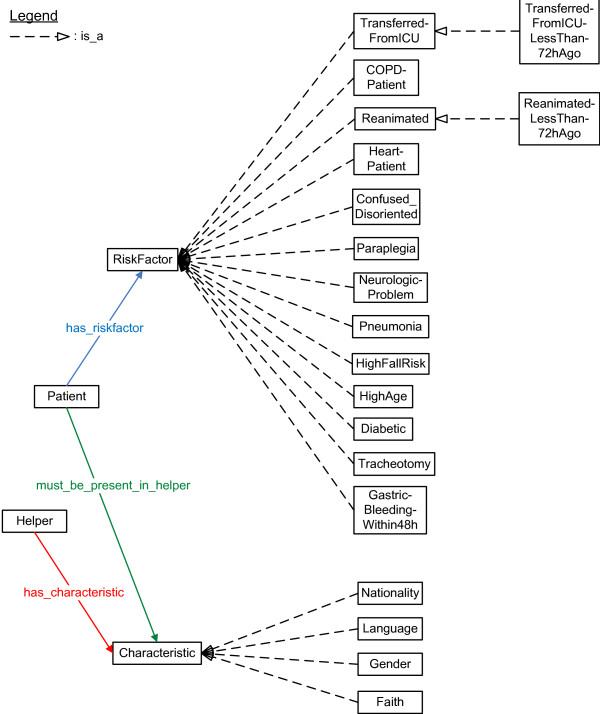
**Fragment of the ontology that models the context information about the characteristics and risk factors**. Fragment of the ontology which models (1) the characteristics of the helpers and (2) the risk factors of the patients. To highlight the possibilities of the system, a (not exhaustive) list of risk factors was assembled by experts from both the medical and nurse call domain. The squares represent the classes. The arrows with the striped lines indicate subclass relationships. The other arrows and lines indicate relations between classes (object properties).

Some characteristics about the helpers are modeled, which can be seen in Figure 4. For the current simulations, the following classes were used: which languages the helpers speak, their gender, their nationality and their religious beliefs. Helpers can indicate patients that they do not want to treat. Patients can then indicate which characteristics they would prefer to be present in the helper that treats them. So, patients cannot directly indicate that they do not want to be treated by a particular helper.

It can be indicated if a patient has one or more risk factors. A complete list of risk factors could be constructed based on a thorough study of the risk factors of patients and the reasons for the calls that they make. Unfortunately, such studies have not been conducted to the knowledge of the authors. To highlight the possibilities of the system, a (not exhaustive) list of risk factors was assembled by experts from both the medical and nurse call domain, as can be seen in Figure 4.

When a patient exhibits a risk factor, he is assigned a probability of belonging to a risk group namely High, Medium and Low Risk Patients. To give a preliminary idea of the benefits of this system, the probabilities were determined by domain experts. At it is difficult to determine exact probabilities for these cases, probabilistic intervals were employed. For example, a diabetic patient has at least 50% chance of being a high risk patient. This is encoded as the probabilistic interval [0.5,1] in the ontology.

Off course patients can have several risk factors, in this case the system will reason over the different probabilities to determine the general probability that a patient belongs to a risk group. This reasoning process is explained in more detail in the *Priority assessment of a call *Subsection of the *Algorithms *Section.

#### Model of the calls and tasks

**Figure 5:**
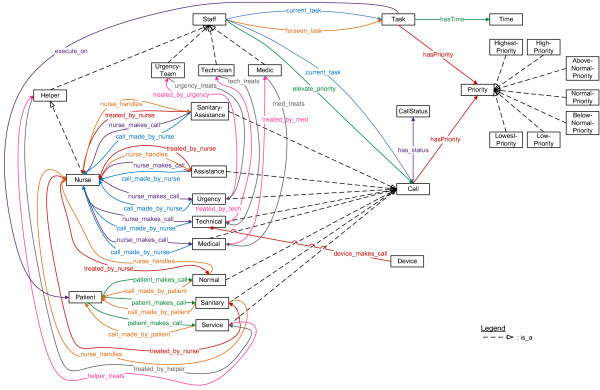
**Fragment of the ontology that models the context information about the calls and tasks**. Fragment of the ontology which models the calls and tasks. It mainly indicates which calls can be made by patients and staff members and which staff members are allowed to handle these calls. Additionally, it models the possible priorities that a calls or tasks can have. The squares represent the classes. The arrows with the striped lines indicate subclass relationships. The other arrows and lines indicate relations between classes (object properties).

Each staff member has an associated current task, as can be seen in Figure 5. For each staff member, it is logged if this staff member is free or busy. Staff members can be handling a call or doing other tasks, e.g. giving medication to a patient. For each task the time by which the task should be completed and the patient for whom this task should be done can be indicated. It is also possible to maintain a list of tasks that a staff member should complete. A task can also be assigned a priority.

A general upper class maintains all the information that is applicable to each call such as the sequence number, the start and end time and the persons who made and handled the call. Each kind of call also has a time-out time. A call can have different statuses. When a call is launched, it has the status Active. This status changes to Answered when a staff member has been called. When the staff member is treating the call, the status changes to Busy. When the job is completely finished, the status is set to Finished.

The different specific calls that can be made are modeled as subclasses of this general upper Call class. For each call it is indicated which kind of person can make the call. As can be seen in Figure 5, three kinds of calls can be launched by patients. A normal call is made for medical problems and a service call is made for a "caring" task. When a normal call is made inside a sanitary room the call is automatically transformed to a sanitary call. All the other calls, namely urgency, medical, technical and (sanitary) assistance calls, are launched by nurses. Which kind of staff member can answer the call is also maintained.

The probabilistic assignment of patients to risk groups is used to determine the priority of the calls. There are seven classes of priorities: Highest, High, Above Normal, Normal, Below Normal, Low and Lowest priority as is illustrated in the upper right corner of Figure 5. The priority of a call is also based on its kind e.g. normal or sanitary. So when a patient from a risk group, makes a certain kind of call, this call is assigned a probability of having a certain priority. For example, when a high risk patient makes a normal call, this call has 2% chance of having a high priority. For now, these probabilities were determined by domain experts at Televic NV. The different devices that can be present inside a hospital also have to be taken into account. Devices such as heart monitors are able to launch technical calls when, for example, their cable is unplugged.

### Algorithms

Several algorithms were constructed to assign the best possible nurse to a call. The first subsection details the algorithm that was used to reason with the probabilistic information to assign a more informed priority to a call that is based on the risk factors of a patient. The second subsection details the algorithm that was used to assign the most suitable nurse to a call.

#### Priority assessment of a call

The general probabilistic information in the ontology about the assignment of patients to risk groups and the priorities of calls can be used to determine the priority of a specific call made by a specific patient. For this the platform needs to reason about the general probabilistic information in the ontology and apply it to the situation at hand.

To model the probabilistic information in the ontology and reason about it, *Pronto *[26] was used. Pronto implements a probabilistic extension of *Description Logics (DLs) *[27], the First-Order Logic on which OWL is based [28]. *Pronto *was chosen because it is easy to use and understand and offers a wide range of reasoning support. All the reasoning is done in a totally logical way without an implicit or explicit translation of the *Knowledge Base *to for example a *Bayesian network*. By using *Pronto*, the probability that a specific call made by a specific patient has a certain priority can be determined. For example, suppose we have a patient, called Patient1, who has two risk factors, namely Diabetes and a Heart disease. Patient1 then makes a Normal call. The ontology contains the probabilistic information (as probabilistic intervals) that a patient with one of these risk factors is a High, Medium and Low Risk patient, as can be seen in Table 1. *Pronto *reasons on this information to conclude that Patient1 has [0.5,1], [0,0.3] and [0,0.1] chance of being a High, Medium and Low Risk patient respectively. The ontology also contains probabilistic information about the probability that a patient from a particular risk group makes a Normal call with a particular priority, as shown in Table 2. *Pronto *combines this information with the previously calculated probability intervals which indicate that Patient1 is a High, Medium and Low Risk patient. *Pronto *concludes that the Normal call of Patient1 has respectively [0,1], [0.1,0.6], [0.3,0.8], [0.1,0.6], [0,1], [0,1], [0,1] chance of having the Highest, High, Above Normal, Normal, Below Normal, Low and Lowest priority.

**Table 1:** The probabilistic assignment of patients to risk groups based on their risk factors

Patient has risk factor	High Risk	Medium Risk	Low Risk
Diabetes	[0.5,1]	[0,0.3]	[0,0.2]
Heart disease	[0.5,1]	[0,0.4]	[0,0.1]

**Table 2:** The probabilistic assignment of calls to a priority category

			Above		Below		
Normal call made by	Highest	High	Normal	Normal	Normal	Low	Lowest
High risk patient		0.2	0.6	0.2			
Medium risk			0.3	0.6	0.1		
Low risk patient				0.6	0.3	0.1	

As shown in the previous example, *Pronto *calculates for each of the seven possible priorities, the probability that the call has this priority. However, one priority needs to be assigned to the call, so this priority can be used in the nurse call algorithm, see *The nurse call algorithm *Subsection of the *Algorithms *Section. To resolve this issue, the following threshold algorithm was employed on the lower bound of the probabilistic intervals. If the probabilistic value for the highest priority class is higher than or equal to the threshold for the highest priority class, it gets the highest priority. If not, the same condition is checked for high, above normal, normal, below normal, low and lowest priority classes. The thresholds can be determined based on the specific characteristics, e.g. number of calls, needs and preferences of the department or hospital. The threshold that were used for the simulations are detailed in the *Collected data *Subsection of the *Evaluation set-up *Section. If the thresholds are 0.21, 0.3, 0.24, 0, 0.05, 0 and 0, ordered from the Highest to the Lowest priority, than the Normal call of Patient1 from the previous example gets the Above Normal priority according to this threshold algorithm.

Although, the 0.2 release of *Pronto *increases the performance of the reasoning tasks over a single probabilistic statement, scalability is still a problem [29]. Currently *Pronto *can handle about 15 probabilistic statements in reasonable time. As a result, *Pronto *cannot currently handle all the probabilistic statements that were added to the ontology in reasonable time.

The following optimization was used in the *oNCS system *to speed up the probabilistic reasoning. First, during down-time, the probabilistic values that indicate that this patient is a high, medium or low risk patient are calculated and stored as known facts in the ontology. This does not have to be repeated often as risk factors do not change a lot during a patients stay in the hospital. Next, when a call is made, all the probabilistic statements that are needed to calculate the priority of this call are extracted from the ontology. Each time, at most 12 probabilistic statements will be extracted, namely the statements about the probabilistic assignment of this patient to the risk groups (3 statements) and the statements about the generic probabilistic assignment of this kind of call to the priority groups (9 statements).

#### The nurse call algorithm

**Figure 6:**
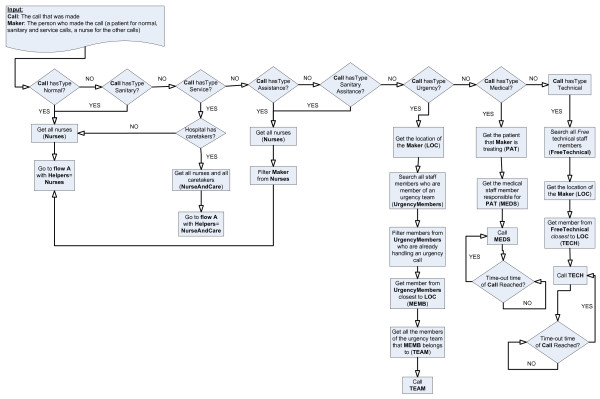
***oNCS *algorithm to find the correct staff member to handle a call**. This figure shows the flow chart of the *oNCS *algorithm, which finds a correct staff member to handle a call. It first determines which kind of calls has been made. Normal, sanitary, service and (sanitary) assistance calls employ the same basic algorithm which is visualized in Figure 7 (Flow A). The difference is that for normal, sanitary and (sanitary) assistance calls only nurses can be called. For service calls caretakers can also be called. It is also made sure that the nurse that made the (sanitary) assistance call, cannot be called to answer this call. Urgency, medical and technical calls each have their own algorithm, which is visualized in this figure.

A new algorithm was designed to find the correct staff member to handle a call. It uses the information stored in the ontology. It first determines which kind of calls has been made as can be seen in Figure 6.

**Figure 7:**
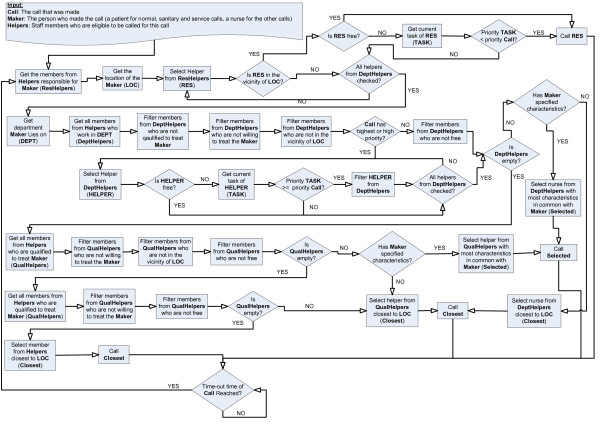
Flow A: *oNCS *algorithm to find the correct staff member to handle a normal, sanitary, service or (sanitary) assistance call

Normal, sanitary, service and (sanitary) assistance calls employ the same basic algorithm which is visualized in Figure 7. The difference is that for normal, sanitary and (sanitary) assistance calls only nurses can be called. For service calls caretakers can also be called. It is also made sure that the nurse that made the (sanitary) assistance call, cannot be called to answer this call.

The common algorithm first checks if the responsible nurse or caretaker can be called. Note that this responsible staff member can also be called if he/she is busy with a task that has a lower priority than the current call. If the responsible nurse or caretaker cannot be called, all the helpers who work on the department where the patient who the call is for lies are investigated. It is assumed that a nurse, who works on a department where the patient lies on, has more background information about the illnesses and concerns of this patient. Only for calls with the highest or high priority helpers are considered that are busy with a task with a lower priority. Otherwise these helpers will never be able to finish the work for the patients they are responsible for. If this option still does not offer a solution, the search is widened beyond the scope of the department and the helpers in the whole hospital are taken into account. If the result is empty again, this means that there are no available nurses in the direct vicinity. The distance becomes a deciding factor at this moment, so the closest nurse with right properties is selected, e.g. free, willing and qualified. If this still does not offer a solution, all the nurses in the hospital are considered and the one who is closest to the patient is called. Note that the characteristics are only used to choose among different available nurses. They are never used to decide that a nurse cannot handle a patient.

The algorithm has a time-out procedure. If a staff member has not indicated that he/she is going to handle the call within the time-out time that is specified for this type of call in the ontology, another staff member is selected to handle the call by running the algorithm again.

Urgency, medical and technical calls each have their own algorithm as can be seen in Figure 6. For urgency calls, the priority lies on finding a person who is near instead of a person who is free. This is necessary because lives are at stake when an urgency call is issued. A time-out procedure is not needed here, as an urgency call will always be immediately answered. The algorithms for the technical and medical calls are rather simple and straightforward because they generally have a very low priority.

Note that a staff member can sometimes be called while he/she is already busy with a task. It is up to the staff member to decide if he/she is going to interrupt his/her current task or not. In contradiction to the place-oriented case, the staff member knows that the new call has a higher priority than the task that this staff member is currently working on. Based on these priorities the staff members can make a more funded decision to interrupt their current task or not. If the staff member decides to answer the new call, the system automatically interrupts the current task of this staff member. If the task is a call, another staff member is searched to handle the call. If it is not a call, the task is added to the list of tasks that this staff member must do. So the staff member does not have to remember himself that he/she has to return to a task or that he/she has to call some other staff member.

### Implementation details

This section gives an overview of the implementation of the entire *oNCS system*. The first Subsection, *Building the ontology*, details how the ontology was digitalized. The second Subsection, *the oNCS platform*, details how the algorithms were implemented and were integrated into the existing *Context-Aware Service Platform (CASP)*.

#### Building the ontology

Different languages exist to digitalize an ontology. The *Ontology Web Language *(*OWL*) [30] was chosen for a number of reasons. First, *OWL *is a recommendation by the World Wide Web Consortium (W3C) [31] and is the most widely used and well-known ontology language. Secondly, using one of the three sublanguage flavors of *OWL*, *OWL-Lite*, *OWL-DL *and *OWL-Full*, one can easily adapt to the required expressiveness at hand. *OWL-DL *is based on *Description Logics *[27], a decidable part of *First Order Logic*. This ensures that reasoning on *OWL-DL *models is computationally complete and decidable, which means that all computations will end in finite time. Thirdly, there also exist a wide range of tools for *OWL *such as editors and visualization tools. Sophisticated *Reasoners *exist that allow checking the consistency and classifying the ontology. *OWL *can also easily be integrated with different *Rule *platforms and can be queried with *SPARQL *[32]. Moreover, *OWL *is the only ontology language for which there exist mature tools to express and reason about probabilistic knowledge. A final advantage is the straight forward integration of an *OWL *ontology into the *CASP framework*, see *the oNCS platform *Subsection, by using *Jena *[33], a *Java *framework for building *Semantic Web *applications.

The *Protégé *editor [34] was used to develop the deterministic part of the ontology. The *Pellet Reasoner *[35] was used to check the consistency and the classification of the ontology. To use the probabilistic *Reasoner Pronto*, probabilistic statements have to be expressed in an *OWL*-file by using axiom annotations, which is a new feature of *OWL 1.1 *[36]. As *Pronto *supports probability intervals, the intervals specified in the *Profile management *Subsection can be used. The exact probabilities were expressed by axioms that were annotated with probability intervals with an equal upper and lower limit.

#### The oNCS platform

The *oNCS platform *was built as an extension of *the Context-Aware Service Platform *(*CASP*) [18]. The *CASP framework *is a collection of bundles for *OSGi *that were developed to handle context information. The *OSGi Framework *[37] is an open service platform for the delivery and control of different applications and services to a certain type of networked device in the environment. In this case the devices would be the portable nurse call buttons, the sensor nodes, the PDAs and the nurse desktop. *OSGi *can best be seen as an application, which is called a bundle in *OSGi*, container. It is possible to plug new bundles into the *OSGi framework *at any time. This expands the framework with new possibilities and services. These new services can be dynamically discovered by the other bundles. So basically, *OSGi *technology provides the standardized primitives that allow applications to be constructed from small, reusable and collaborative components. The open source implementation *Knopflerfish *was used.

**Figure 8:**
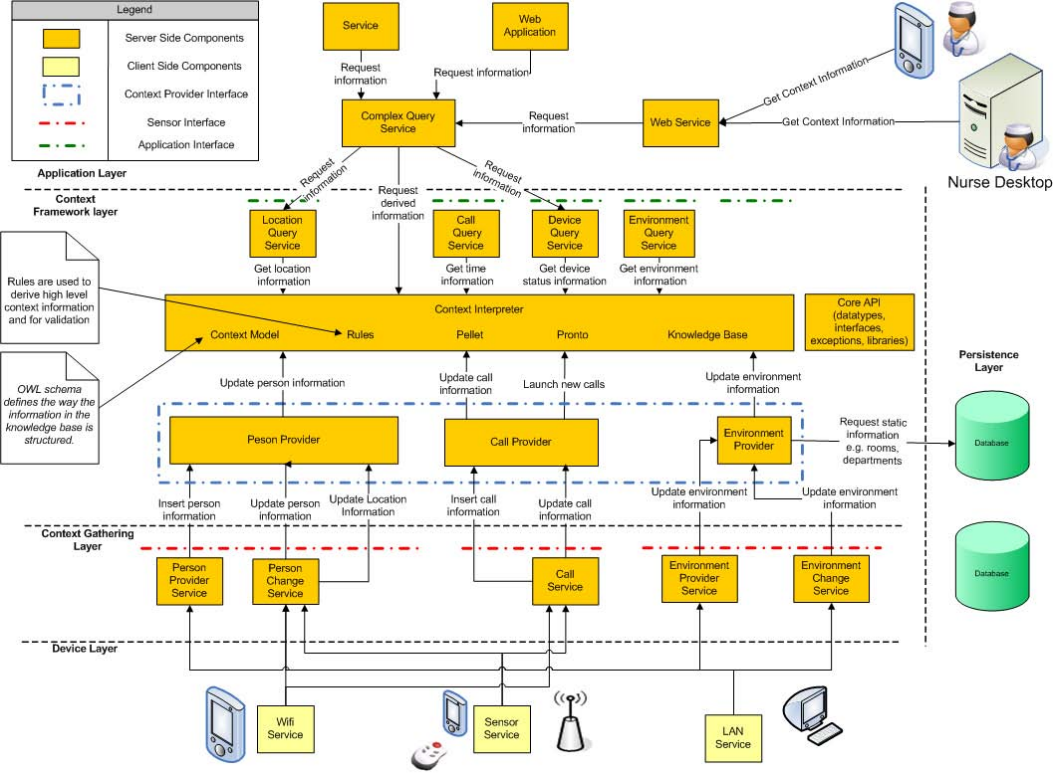
**The architecture of the *oNCS platform***. This figure represents the architecture of the *oNCS *platform. The *Context Framework Layer *is the most important layer. Within this layer the *Context Interpreter *controls all the context information. The ontology determines the structure of the *Knowledge Base*. The *Knowledge Base *contains all the data that conforms to the ontology. The *Context Model *provides access to the ontology by using *Jena*. *Pellet *is used to check the consistency of the model. The layer also holds all the *Rules *that work with the information in the *Knowledge Base*. The different *Context Providers *allow importing external information into the framework. This information is then added to the *Knowledge Base*. This new information can come from a database (*Persistence Layer*) or directly from a device (*Device Layer *and *Context Gathering Layer*). Currently three *Context Providers *are provided: the Person Provider, the Environment Provider and the Call Provider. The *Query Services *are used to extract information from the *Knowledge Base*. The *Query Services *can be used to visualize the knowledge or to use the information in another application (*Application Layer*). The methods in the *Context Providers *and *Query Services *were also made available as *Web Services*.

An overview of the *oNCS platform *is shown in Figure 8. The *Context Framework Layer *is the most important layer. Within this layer the *Context Interpreter *controls all the context information. The ontology determines the structure of the *Knowledge Base*. The *Knowledge Base *contains all the data that conforms to the ontology. The *Context Model *provides access to the ontology by using *Jena*. *Pellet *is used to check the consistency of the model. The layer also holds all the *Rules *that work with the information in the *Knowledge Base*.

The different *Context Providers *allow importing external information into the framework. This information is then added to the *Knowledge Base*. For example, the *Person Provider *is used by the sensor nodes to insert new information about the location of the patients and staff members. This new information can come from a database (*Persistence Layer*) or directly from a device (*Device Layer *and *Context Gathering Layer*). Currently three *Context Providers *are provided: the Person Provider, the Environment Provider and the Call Provider. All the *Context Providers *implement a common interface, namely ContextProvider, which makes it easy to plug new *Context Providers *into the framework.

The *Query Services *are used to extract information from the *Knowledge Base*. This ensures that application developers do not have to write the error-prone queries themselves. They also do not have to translate the results of the queries to usable *Java*-objects. The *Query Services *can be used to visualize the knowledge or to use the information in another application (*Application Layer*).

To make the platform more generic some *Web Services *were developed. These *Web Services *allow applications and devices from anywhere in the network to call methods to add new information to the *Knowledge Base*, such as making new patients, nurses or calls, or extract information, such as which nurse has been called to answer a call. These methods call the *Context Providers *and *Query Services *to add or extract the knowledge.

Note that the framework is modularly divided into bundles. These bundles can be plugged into the *Knopflerfish *(*OSGi*) framework and can dynamically discover each other. This also allows deploying the framework in a distributed manner, which is important when high performance is needed. The *oNCS platform *runs on multiple servers to ensure reliability and scalability. When a server goes down, another server can still process all the requests. Standard load-balancing algorithms [38,39] can also be used to distribute the requests amongst the different servers.

To improve the scalability of the system, information that is no longer needed in the ontology can be stored in a database so it can be used for studies or analysis. This can for example be done at night. A lot of information can be removed from the ontology each day such as calls that have been completely handled or patients that have left the hospital. The server additionally also logs all the actions of the systems such as who added which information to the ontology, which calls were launched and who handled them.

The *oNCS *nurse call algorithm is implemented by using *Rules*. The *Rules *are activated when an event occurs in the *Knowledge Base *for example when a new call is added. When the condition is fulfilled, the *Rule *calls a *functor*. A *functor *does some calculations with the parameters it receives from the *Rule*, for example the new call. The *functor *can also change the information in the *Knowledge Base*.

Every kind of call that can occur is handled by a different *Rule*. For example, the following code fragment shows the *Rule *that reacts to a normal call:

[insert_nurse_normalcall:

(?x rdf:type ncs:Normal)

(?x ncs:has_status ?CallStatus)

(?CallStatus ncs:Kind 'Active')

noValue (?x ncs:treated_by_nurse)

→ findHelper (?x) ]

As can be seen, this *Rule *is activated when a normal call is launched (its status is Active and no staff member has been called). If the condition is fulfilled the *functor *findHelper() is called which takes the call as argument. The *functor *follows the earlier stated algorithm specified in *The nurse call algorithm *Subsection of the *Algorithms *Section to find a correct staff member to handle the call. It adds the information that this particular staff member has to handle this particular call to *Knowledge Base *(the treated_by relation in the ontology). This guarantees that the *Rule *is not fired again, because the noValue condition is no longer fulfilled. All the other types of calls are handled in a similar matter.

*Rules *were also constructed that trigger when the status of a call is changed. The Rules adapt the Knowledge base for example to indicate that a nurse is busy with a call, has finished a call, the time at which the call was finished and so on. Most importantly these Rules also automatically interrupt the current task (if any) of the called staff member as explained in *The nurse call algorithm *Subsection of the *Algorithms *Section. A last set of *Rules *is used to implement the time-out procedure for each kind of call.

Note, that if a different nurse call algorithm should be used, e.g. because another hospital might use a different nurse call policy, only the *functor *needs to be rewritten. This can be easily done as a lot of re-usable methods and code have been provided e.g. to collect the needed information from the ontology, compare the preferences of the patient with the characteristics of the staff members or find the closest staff member.

### Evaluation set-up

To test and demonstrate the advantages of the new *oNCS platform*, simulations were set up with realistic data about a nursing department of the Ghent University Hospital [19].

#### Collected data

**Figure 9:**
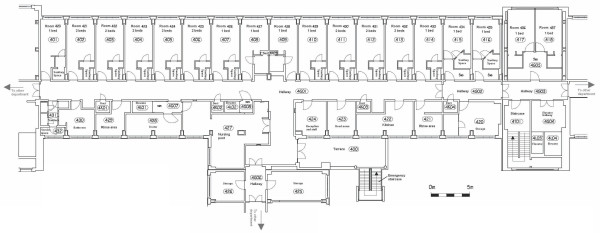
**The floor plan of the studied department**. This figure represents the floor plan of the studied department of the Ghent University Hospital. The department contains patients that are fairly mobile. The most important spaces to notice on the floor plan are the rooms and the sanitary areas. The department contains 26 beds. The floor plan indicates for each room how many beds it contains. Most rooms have their own sanitary space, but there are also some shared sanitary spaces. The nursing post is the place where nurses reside when they are not helping patients. This space is used to for example prepare medication or write reports. The head nurse has her own office. Patients do not have access to the storage and service spaces, the terrace, the rinse areas and the kitchen. The doors on the left and right of the floor plan are used to go to other departments. Generally patients use the elevator on the right of the floor plan to leave the department. The elevator in the middle of the floor plan is generally only used by staff members.

The studied department of the Ghent University Hospital contains patients that are fairly mobile. They are not confined to their beds, but they do spend most of their time in their room. The most important mobile activities are going to the restaurant, going outside to smoke, getting the newspaper and being moved to other departments to undergo some additional medical examinations. The floor plan can be seen in Figure 9. The most important spaces to notice on the floor plan are the rooms and the sanitary areas. The department contains 26 beds and has an occupation rate of 84.62%. Each room has one or 2 beds.

The three most visited spaces by patients were included in the simulations, namely the smoking area just outside the building, the CT scanner and the cafeteria at the ground-level of the building. The time it takes to travel to all these spaces from the department was measured. Finally, it was determined how patients divide their time over these different spaces.

Some information about the staff in this department was also gathered. The department has three shifts: the early, late and night shift. During the week there are 5 nurses during the early shift, 4 during the late shift and 1 or 2 during the night shift. During the weekend there are 4 nurses during the early shift, 3 during the late shift and 1 or 2 during the night shift. The department also has a head nurse, but this head nurse never answers calls. Each nurse is responsible for approximately 5 or 6 patients during a shift. They are assigned based on the split-up of the rooms of the department according to the number of present nurses. Patients in adjacent rooms are assigned to the same nurse. A patient is never assigned to more than one nurse at the same time.

**Figure 10:**
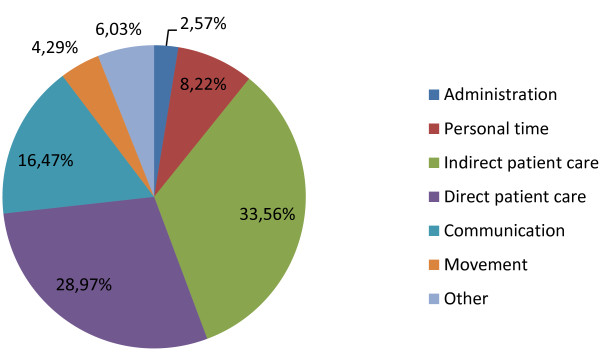
Distribution of time of the nurses across different kinds of tasks

The walking behavior of the staff members was simulated by using information, which was gathered during an earlier study [40], about their tasks and the percentage of time they spend on each group of tasks, as visualized in Figure 10. For each of the tasks it was also determined if the task was always (low or lowest priority), never (high priority) or sometimes interruptible (below, above or normal priority).

To monitor the added value of keeping the characteristics in the ontology, information was gathered about the spoken languages by both the patients and the staff members. All the staff members are able to speak Dutch, 80% of the staff members speak English, 70% speak French, 20% speak German and none of them speak Italian or Spanish. On the other hand, 2% of the patients only speak French and 3% of them only speak German.

It was determined how many patients have none, 1, 2 or more risk factors and which risk factors were more frequent than other risk factors by assigning a weight to them, as can be seen in Table 3. Some combinations of risk factors were deemed to be more frequent than others:

• COPD and tracheotomy

• High age and disoriented/confused

• High age and high fall risk

• Diabetes and disoriented/confused

• Neurological problem and disoriented/confused

• Transferred from the ICU and tracheotomy

Based on this data, patients were assigned risk factors.

**Table 3:** The distribution of the risk factors amongst patients in the three departments

**Number of patients with**:	
0 risk factors	10
1 risk factor	10
2 risk factors	8
> 2 risk factors	2
**Risk factor weights (%)**:	
High age (a)	50
Diabetes (b)	10
Heart disease (c)	3
High fall risk (d)	5
Neurologic problem (e)	3
Tracheotomy (f)	10
COPD (g)	3
Paraplegia (h)	3
Pneumonia (i)	3
Disoriented/confused (j)	5
Gastric Bleeding within 48 h (k)	3
Transferred from ICU (l)	1
Transferred from ICU within 72 h (m)	0
Reanimated (n)	1
Reanimated within 72 h (o)	0

The thresholds for the probabilistic reasoning algorithm, see the *Priority Assessment of a call *Subsection of the *Algorithms *Section, were determined by generating 22500 realistic calls and determining the priority each call gets by adjusting the threshold. The combination of thresholds was searched for which the percentages of calls assigned to a certain priority deviated least from the following distribution: 5% calls with highest priority, 10% with high priority, 25% with above normal priority, 35% with normal priority, 25% with below normal priority and 0% with the low and lowest priority. This distribution reflects a realistic hospital environment. The tested kinds of calls generally do not have the low or lowest priority as these categories are preserved for medical and technical calls. The middle categories, namely above normal, normal and below normal, generally contain more calls as most calls are made for simple requests. The chosen thresholds are 0.21 for the highest priority, 0.3 for the high priority, 0.24 for the above normal priority, 0 for the normal priority, 0.05 for the below normal priority and 0 for the low and lowest priority.

#### Current nurse call algorithm

When calls are made by patients inside rooms, they are treated as normal calls. When calls are made inside a sanitary space, they are treated as sanitary calls. Nurses are able to make (sanitary) assistance calls in this department, but there are no buttons to make urgency or medical calls. Technical calls are not taken into account in the simulations as the result is straight-forward. Technical calls always get the lowest priority and a member of the technical staff is called as explained in *The nurse call algorithm *Subsection of the *Algorithms *Section.

**Figure 11:**
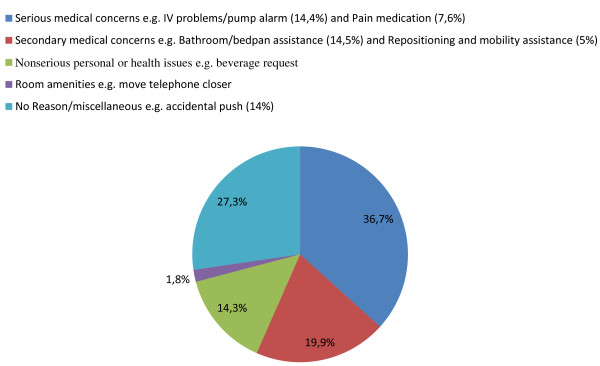
**Reasons for patients' call light use **[41].

Information about the calls, such as frequency and duration, was gathered during three weeks by studying the logging information of the currently installed place-oriented nurse call system. Limited research has been done on reasons for patients' call light use in the Ghent University hospital. The paper by Meade [41] presents an extensive study about this subject. The used results are presented in Figure 11. When a call is made a reason is randomly assigned based on these percentages. The average time that a nurse spends on handling a task from each category was also determined in an earlier study [40].

The normal, sanitary and (sanitary) assistance calls are handled as follows. All the nurses of the department receive the calls on their beepers or portable phones. A light also switches on above the room of the patient. The nurse, who arrives first at the location of the patient, switches off the call and starts treating the patient. If the time-out of a call is reached and none of the staff members have come to handle the call, all the nurses of the department receive the call again. As can be seen, it is possible that multiple nurses arrive at a room to handle a call, as multiple nurses are called and one nurse does not know if another nurse will handle the call or not. If they interrupt their current task (which could also be a call) to handle this call, the nurses have to remember themselves that they have to go back to that interrupted task. In case of an interrupted call, the other patient also has to wait until the nurse has finished this call, while it could be of a lower priority.

#### Simulation set-up

A realistic day-to-day hospital scenario was simulated. This means that the beds in the department are occupied averaging around the occupation rate as indicated in the *Collected data *Subsection. Of course it is assumed that the patients already own portable buttons and can thus move around freely and still make calls. When this situation is simulated for the place-oriented system, some calls may be impossible to handle e.g. calls made in the middle of a hallway. The movements of the patients were determined out of the collected data about the mobility of the patients and their tendency to visit other areas. During these movements they can make (sanitary) calls modeled according to a Poisson process with λ = 0.001164021. Once a patient makes a call, it is assumed that this patient stands still. The movements of the nurses were determined out of the collected data about how they divide their time around their different kinds of tasks. During these tasks they receive calls of patients. They will only interrupt their current task, if the call has a higher or equal priority. They will only interrupt current calls, if the new call has a higher priority. If the new call does not have a priority, as can occur in the place-oriented system, a nurse chooses randomly to interrupt his or her current task or call. During the handling of a call, nurses will launch a (sanitary) assistance call with a probability of 0.07386%. If a nurse has to choose between multiple calls to handle, it is assumed that the nurse chooses the one with the highest priority. If the calls do not have priorities or multiple calls have the same priority, the closest call is chosen. It is assumed that patients or nurses that are on the move advance 1 meter in the direction of their goal during each time step. Characteristics of patients and nurses, risk factors of the patients and responsibility of staff members for certain patients were simulated as indicated in the *Collected data *Subsection.

The simulation was done 30 times for each of the 3 shifts during the weekend and 30 times for each of the 3 shifts during the week. These simulations were done on a PC with the following specifications: Intel Core 2 Duo Processor P8600 (2.40 GHz, 1066 MHz, 3 MB), 4 gigabyte of RAM (2 × 2 gigabyte) and a 250 GB Serial ATA (7200 RPM) hard drive.

## Results

Both the *oNCS *and the place-oriented system were simulated in a realistic hospital setting. The first subsection details the results of the comparison between the two. The advantages of the probabilistic risk assessment algorithm were also evaluated. Finally, the performance of the system is discussed.

### Simulation Results

As mentioned in the *Current nurse call algorithm *Subsection of the *Evaluation set-up *Section, it is possible that multiple nurses arrive at a room to handle a call in the place-oriented system. On average 0.43 unnecessary nurses arrived at a call per simulation, with a maximum of 4 nurses in 1 simulation. This means that, at least one nurse each day arrives at a call which is already being treated by another nurse.

As mentioned in the *Simulation set-up *Subsection of the *Evaluation set-up *Section, some calls are impossible to handle in the place-oriented system as they are made in a place, e.g. the hallway, where currently no buttons are provided. These can be handled by the *oNCS system *as the patients have portable buttons. On average 2.53 impossible calls were made per simulation, with a maximum of 12 impossible calls in 1 simulation. This means that each shift about 3 calls cannot be handled in the current system. Especially the worst case scenario with 12 impossible calls is alarming. Patients can walk around in the hallways and staircases and are unable to make calls. Especially outside in the smoking area there are no staff members close, who could help the patient fast.

**Figure 12:**
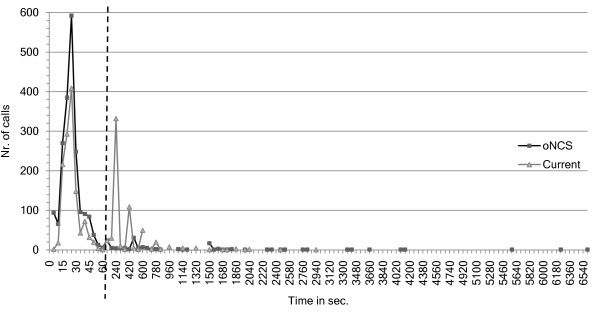
**Number of calls as a function of the nurse arrival times**. This figure shows the number of calls that have a nurse present (y-axis) as function of the arrival times of these nurses (x-axis in seconds) for both the *oNCS system *and current, place-oriented system. This means that the nurse has arrived at the place where the patient made the call. Note that the first part of the x-axis has a time-step of 5 seconds, while the second part has a time-step of 60 seconds. The two parts are separated by the striped vertical line.

Figure 12 shows the number of calls that have a nurse present as a function of the arrival times of these nurses. This means that the nurse has arrived at the place where the patient made the call. Note that the first part of the x-axis has a time-step of 5 seconds, while the second part has a time-step of 60 seconds. Most of the calls have a nurse present after 60 seconds in the *oNCS system*. In the place-oriented system about half of the calls have a nurse present after 60 seconds. Most of the rest of the calls are handled after 780 seconds.

The difference can be easily explained. In the *oNCS system *only one nurse receives the call. In most cases the call will have a higher priority than the current task of the nurse because the algorithm takes this into account. Therefore, the nurse will immediately go and answer the call. In most cases the distance to the patient will be limited, as this is taken into account in the novel nurse call algorithm.

On the other hand in the place-oriented system, multiple nurses receive the call. They have to decide if they are going to quit their current task. They have to make this decision without information about the call. So in the case that all nurses ignore the call, thinking someone else will handle it, the call has to be relaunched before it is noticed that nobody went to handle the call. This is illustrated nicely on the graph, as a peak can be seen each time the call is relaunched, namely shortly after 180, 360, 540,... seconds. Moreover, the distance is not taken into account when calling the nurses in the place-oriented system. So it is possible that the nurse must walk a long time before arriving at the room of the patient.

The tail of the *oNCS system *is much longer than the place-oriented system. This is caused by the impossible calls which are not answered in the place-oriented system, but which are answered in the *oNCS system*. Most of these calls occur in places that are very far away from the department e.g. normal calls in the smoking area and restaurant or assistance calls in the scanner room. This could be solved by allowing nurses from closer departments to answer these calls. However, these nurses were not included in the simulations.

**Figure 13:**
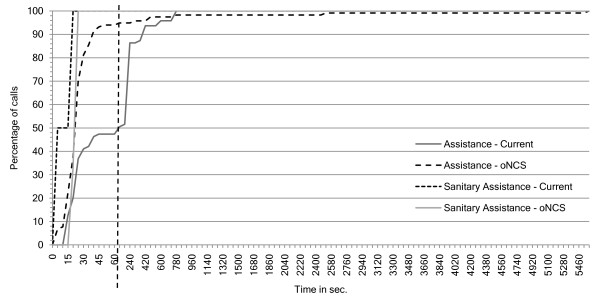
**Percentage of (sanitary) assistance calls as a function of the nurse arrival times**. This figure shows the percentage of assistance and sanitary assistance calls (y-axis) as function of the arrival times of these nurses (x-axis in seconds) for both the *oNCS system *and the current, place-oriented system. This means that the nurse has arrived at the place where the patient made the (sanitary) assistance call. Note that the first part of the x-axis has a time-step of 5 seconds, while the second part has a time-step of 60 seconds. The two parts are separated by the striped vertical line.

As can be seen in Figure 13, 100% of the sanitary assistance calls have a nurse present within 15 seconds in both the current and *oNCS system*. This is because these calls generally have a very high priority. The *oNCS system *is slightly slower than the place-oriented system which can be explained by the initial delay of calling the nurse call algorithm (see the *platform performance *Subsection). However, for the assistance calls the *oNCS system *performs much better than the place-oriented system. In the *oNCS system *95% of the assistance calls have a nurse present within the first minute. In the place-oriented system this only occurs after 480 seconds (8 minutes).

**Figure 14:**
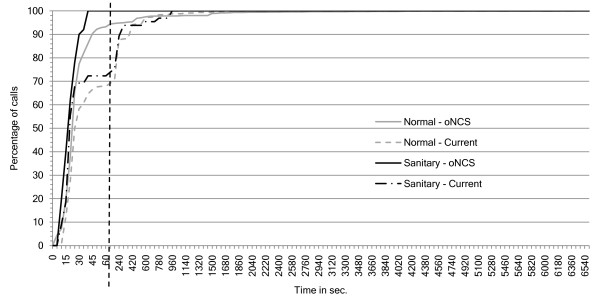
**Percentage of normal and sanitary calls as a function of the nurse arrival times**. This figure shows the percentage of normal and sanitary calls (y-axis) as function of the arrival times of these nurses (x-axis in seconds) for both the *oNCS system *and the current, place-oriented system. This means that the nurse has arrived at the place where the patient made the normal or sanitary call. Note that the first part of the x-axis has a time-step of 5 seconds, while the second part has a time-step of 60 seconds. The two parts are separated by the striped vertical line.

A similar scenario can be spotted for the sanitary calls in Figure 14. In the *oNCS system*, 100% of sanitary calls have a nurse present after 40 seconds. In the place-oriented system, only 72% of the sanitary calls are handled at this point and takes 960 seconds (16 minutes) until all the sanitary calls have a nurse present. The normal calls generally also have a nurse present faster in the *oNCS system*. 90% of these calls have nurse present within 45 seconds. In the place-oriented system only 66% of the calls have a nurse present then. It reaches 90% after 300 seconds (5 minutes). A small percentage of these calls take a long time to be handled, notably for the assistance calls and normal calls in the *oNCS system*. This can again be explained by the impossible calls which are answered in the *oNCS system*, but not in the place-oriented system.

**Figure 15:**
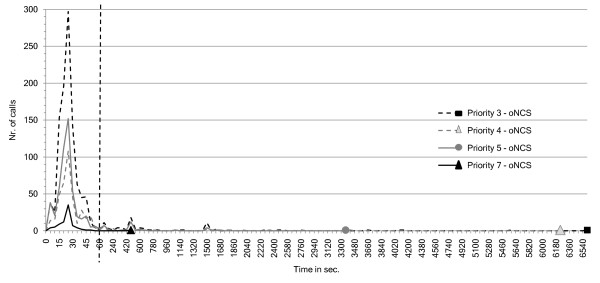
***oNCS system*: number of calls as function of nurse arrival times for different call priorities**. This figure visualizes the number of calls that have a nurse present (y-axis) as function of the arrival times of these nurses (x-axis in seconds) for different call priorities for the *oNCS system*. This means that the nurse has arrived at the place where the patient made the call. This allows evaluating (1) the influence of the priority of the call on the arrival time of the nurse (2) the distribution of the calls amongst the different priorities. Note that the first part of the x-axis has a time-step of 5 seconds, while the second part has a time-step of 60 seconds. The two parts are separated by the striped vertical line.

**Figure 16:**
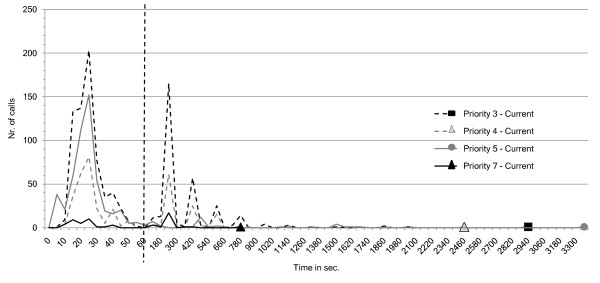
**Place-oriented system: number of calls as function of nurse arrival times for different call priorities**. This figure visualizes the number of calls that have a nurse present (y-axis) as function of the arrival times of these nurses (x-axis in seconds) for different call priorities for current, place-oriented system. This means that the nurse has arrived at the place where the patient made the call. This allows evaluating (1) the influence of the priority of the call on the arrival time of the nurse (2) the distribution of the calls amongst the different priorities. Note that the first part of the x-axis has a time-step of 5 seconds, while the second part has a time-step of 60 seconds. The two parts are separated by the striped vertical line.

The number of calls that have a nurse present as a function of the arrival times of these nurses for different call priorities are visualized in Figure 15 for the *oNCS system *and in Figure 16 for the place-oriented system. As can be seen the distribution of the calls amongst the different priority levels is as to be expected. The below normal priority is assigned the most. This department contains a considerable amount of patients without any risk factors, when they make a normal call it will get the below normal priority. Moreover some patients with a minor risk factor would also make normal calls that get this priority. The normal and above normal priorities are assigned to a comparable amount of calls. These are for example sanitary calls or calls made by patients with some risk factors. The highest priority gets assigned to the least amount of calls. These are primarily sanitary assistance calls or assistance calls made by patients with some risk factors.

The amount of time it takes for a nurse to be present after the call is made in the *oNCS system *is also as expected. Calls with the highest priority are handled the fastest. Most of those calls have a nurse at the scene within 45 seconds. The worst case scenario still has a nurse at the scene within 480 seconds. The calls with below normal, normal and above normal priorities are handled somewhat slower but most calls still have a nurse in place within 60 seconds. The tails are longer, but still in the correct order: the worst-case time of the calls with above normal priority is lower than the worst-case time of calls with the normal priority which is in turn lower than the worst-case time of the calls with the below normal priority.

However, the amount of time for a nurse to be place is not as logical in the place-oriented system. It is obvious that the place-oriented system does not take the priority of the call into account. The different peaks can be explained by the relaunch times of the calls. Every 180 seconds a call which does not have a nurse in place is relaunched. It can be seen that after these time points (0, 180, 360,...) a series of calls is answered. Even calls with the highest priority need to be relaunched up to 4 times before someone is in place. The calls with the above normal, normal and below normal priority have the same trend of having a nurse in place within a certain time. Only calls with the above normal priority seem to be handled faster than the other calls. However, the calls with this priority also have the longest worst-case time.

Table 4 gives an overview of the distribution of calls amongst the nurses present in the department. The first column indicates the number of nurses that were present in the department during the simulation. For both systems it is shown what the maximum and minimum percentage of calls was that a nurse handles during a shift. It is also indicated how many nurses handle zero calls during a shift. Finally, the standard deviation is given between the percentage of calls that nurses handles and the mean. The mean is of course the ideal percentage of calls that a nurse should handle, for example 50% in the case there are 2 nurses in the department. Note, that the number of calls that a nurse handles per shift is different from the amount of calls that this nurse is assigned and thus receives on the portable phone or beeper.

**Table 4:** Distribution of calls amongst the nurses

Nr. of nurses in the department	Workload distribution				Workload distribution			
	Place-oriented system:				*oNCS system*:			
	Max.	Min.	# 0%	Std. Err.	Max.	Min.	# 0%	Std. Err.
1	100	100	0	0	100	100	0	0
2	70.97	29.03	0	11.90	57.14	42.86	0	5.12
3	58.33	12.12	0	12.65	60.98	6.98	0	14.74
4	62.50	0	1	12.26	50	0	1	10.24
5	54.84	0	2	12.63	42.50	2.56	0	7.90

The *oNCS system *leads to a slightly better workload distribution than the place-oriented system. Especially in the case when there are only 2 nurses available in the department all the calls are divided more evenly amongst the different nurses. In the *oNCS system*, all the nurses get about 50% of the calls, while in the place-oriented system some nurses get up to 70% of the calls (while the other nurse in the department at that time thus only gets 30% of the calls). The difference is also obvious in the case of four and five nurses as the extremes are made less extreme in the *oNCS system*.

### Platform performance

#### Performance of the probabilistic reasoning

As mentioned in the *Priority Assessment of a call *Subsection of the *Algorithms *Section, the implementation was optimized to cope with the insufficient scalability of the probabilistic reasoning. The optimization ensures that at most 12 probabilistic statements will be extracted from the ontology on which probabilistic reasoning needs to be performed. The measurements were done using *Pronto *probabilistic *Reasoner *0.2 on a computer with the same specifications as the previous section.

The averages and confidence intervals of all the measurements of the reasoning tasks on the ontology with 12 probabilistic statements can be seen in Table 5. First, the consistency and the satisfiability of the ontology were checked. Next, the performance of entailing some probabilistic statement on concept (*T-Box*) level that was explicitly stated in the ontology was checked. The performance of entailing a probabilistic statement on concept (*T-Box*) level that was not explicitly stated in the ontology was derived. *Pronto *would have to reason about the probabilistic statements to find the correct probabilistic interval. Finally, a probabilistic statement on instance (*A-Box*) level, which was explicitly stated in the ontology, was entailed. The performance is always below 4 seconds, which is acceptable.

**Table 5:** Performance measurements of the probabilistic reasoning tasks on an ontology with 12 probabilistic statements

Probabilistic Reasoning Task	Average (ms)	CI 95%	CI 99%
Consistency	2165.43	91.18	119.83
Satisfiability	473.80	5.02	6.59
Entail Generic Stated	3030.87	107.73	141.58
Entail Generic Unstated	3995.70	82.26	108.11
Entail A-Box Stated	2508.80	38.16	50.15

#### Performance of the nurse call algorithm

Table 6 visualizes the performance of the different parts of the nurse call algorithm, namely assigning a staff member to a call and answering, treating (change status to busy) and finishing a call. Note that these results do not take into account the probabilistic reasoning to determine the priority of the call. As mentioned in the previous section, this reasoning was done in advance. When a call is launched, a suitable nurse is notified within 50.333 ms on average, which is a negligible delay.

**Table 6:** The performance results of the nurse call algorithm

Call & algorithm	Average time (ms)	CI-95%	CI-99%
**Normal call**:			
Assign nurse	42.38	0.53	0.69
Answer call	49.79	0.50	0.65
Treat call	12.78	0.25	0.33
Finish call	65.07	0.55	0.72
Relaunch call	24.27	0.18	0.23
**Sanitary call**:			
Assign nurse	49.87	2.87	3.78
Answer call	54.47	3.36	4.42
Treat call	16.17	1.67	2.19
Finish call	66.71	3.97	5.21
Relaunch call	31.24	0.86	1.13
**Assistance call**:			
Assign nurse	57.33	2.72	3.58
Answer call	58.44	3.46	4.55
Treat call	13.18	1.57	2.07
Finish call	68.07	3.59	4.71
Relaunch call	/	/	/
**Sanitary assistance call**:			
Assign nurse	68.25	30.96	40.69
Answer call	54.63	12.80	16.82
Treat call	11.88	5.09	6.68
Finish call	52.63	39.11	51.40
Relaunch call	/	/	/
**Urgency call**:			
Assign nurse	33.83	8.69	11.42
Answer call	103.07	8.39	11.03
Treat call	7.40	3.54	4.65
Finish call	139.40	6.20	8.15
Relaunch call	/	/	/

## Discussion

The first observation is that maintaining the profile information of the patients and the staff members leads to a lot of advantages.

The novel nurse call algorithm takes this information into account to intelligently assign nurses to handle calls. The place-oriented algorithm only considers which patients (actually rooms) are allocated to which nurses. In the new algorithm much more factors are taken into account. It considers the characteristics and the status of the staff members, the risk factors and preferences of the patients, the priority of the call and so on.

The nurse is able to track the location of the patient who made the call (location-awareness). Additionally the nurse also knows which patient made the call. In rooms with multiple patients, it is impossible to know accurately who made the call in the place-oriented system. In the *oNCS system*, the nurse knows specifically which patient made the call and can use this information to determine if he/she is going to answer the call or not, if medication or equipment will be needed and so on.

Even when the patient does not make a call, the nurse can access a lot of information about the patient on her PDA such as the risk factors of the patients, his or her room number and so on.

The nurse can also use the PDA to collect information about the other staff members such as their locations, if they are busy or free, which priority their task has and so on. This also makes is easier to determine if he/she is going to handle a call or not. Nurses can indicate that they are going to answer a call. In the place-oriented system unnecessary nurses are often called, which means that multiple nurses arrive at a room of a patient to handle the call. This leads to unnecessary interruptions of other tasks by these nurses. Moreover, only one nurse is called in the *oNCS system *to handle a call, while in the place-oriented system multiple nurses are often called. Additionally, it has been shown that a lot of time in hospitals is spent on trying to find someone. This will also be reduced by employing the *oNCS system*.

When a call is assigned to a nurse in the *oNCS system*, the nurse is certain that he/she is in the vicinity of the patient. Nurses that are too far away are not called to handle a call. In the place-oriented system, a nurse is sometimes called when he/she is very far away from the patient as the distance is not taken into account. The nurse cannot be sure that someone else will handle this call, which means that this nurse will have to turn back to answer the call.

When a task is interrupted, the nurse does not have to remember himself/herself that he has to return to it. The *oNCS system *does this for the nurse. This leads to fewer forgotten tasks and lesser work pressure on the staff.

A disadvantage of maintaining the profile information is the overhead that is introduced by the fact that all this information about the patients and staff members has to be inputted into the computer. Therefore this task has to be supported by a very user-friendly interface.

Secondly, the novel nurse call algorithm also leads to significant measurable improvements in the manner nurses are assigned to calls. The novel nurse call algorithm leads generally to a better workload distribution amongst the nurses as it takes into account the current task of the nurse and its priority. Additionally, only one nurse is called to handle a call, which prevents that multiple nurses arrive at a patient to handle the call. Because of this patient generally are treated quicker than in the place-oriented system. This is also caused by the fact that the distance is taken into account when searching a nurse to handle a call. Moreover, the novel nurse call algorithm takes the kind and priority of the call into account. Calls with a higher priority are generally handled faster than calls with a lower priority. This is not the case in the place-oriented system. Moreover, (sanitary) assistance calls are also generally handled faster than normal and sanitary calls. This is achieved because when a nurse receives a call while this nurse is performing a task (or even handling another call), the nurse is sure that the new call has a higher priority. This way the nurse can make a more well-funded decision on whether he/she is going to interrupt the current task or not. Moreover, the nurse is more likely to interrupt his/her task as he/she knows that this call has a higher priority and he/she is the most appropriate nurse to handle this call at this moment.

The performance of the novel nurse call algorithm is very good. A suitable nurse is notified within 50.333 ms on average, which is a negligible delay. This means that the general guidelines outlined by some countries can still be achieved. These guidelines stipulate that at least one staff member should arrive at the location of the patient within 3 minutes when an urgency call was made and within 5 minutes for normal, sanitary, service and (sanitary) assistance calls. The achieved performance does not endanger meeting these requirements.

The system scales up to at least 30 patients and 20 nurses. Thus, a lot of profile information can be retained without decreasing the performance of the system. Large-scale simulations need to be performed to profile the complete scalability of the system.

Thirdly, the portable buttons improves the mobility and the safety of the patients. Patients can walk around freely and are still able to make calls. As can be seen in the simulations it often occurs that patients need to make calls in remote areas such as smoking areas or the restaurant, where there are no nurses present. This problem is of course most prominent in departments where patients are fairly mobile e.g. the patients spend at least 10% of their time walking around.

Finally, the dynamic priority assessment of calls instead of statically defining these priorities provides a number of advantages. The priority of a call depends on the risk factors of the patients and the kind of call. This means that the priority of a call is adjusted to the specific needs and profile of the patient. This leads to a wider range of priorities of the calls that are made. This means that a patient can make calls with varying priorities depending on the current risk factors of the patient and the kind of call.

The scalability of this probabilistic assessment was presented in the *Platform Performance *Subsection of the *Results *Section. These results can be improved by calculating the probabilistic values that indicate that the patient is a low, medium or high risk patient during down-time for example at night. These are stored as facts in the ontology. This procedure does not have to repeated often as most risk factors of a patient tend not to vary that much. After doing this, the number of probabilistic statements to determine the priority of a call of a specific patient is significantly reduced to achieve an acceptable performance.

However, our study also has some limitations. A first limitation is that the probabilities in the ontology were only determined by domain experts. These probabilities indicate the probability that a patient belongs to a certain risk group based on the risk factors of this patient. A complete list of risk factors and accompanying probabilities could be constructed based on a thorough study of the risk factors of patients and the reasons for the calls that they make. However, this study is not yet conducted as the goal was to give an idea of the benefits of incorporating probabilistic priority assessment in the *oNCS system*. Probabilities were also added to the ontology to express the probability that a call of a particular kind made by a patient from a particular risk group has a particular priority. These probabilities were also determined by domain experts. In the future, the *oNCS system *could automatically learn and adapt these probabilities based on logging data from the *oNCS system*. This would make the *oNCS system *self-learning.

A second limitation is that the system has not been deployed in a real life environment yet. Our results are purely based on simulations. Nevertheless, these simulations were based on realistic data obtained from a department of Ghent University Hospital. However, no real observations were done in this department. The data was gathered by questioning the staff who works at the department and by examining the logging data of the current place-oriented nurse call system used in the department. This data gives us clear picture of how the patients and staff members currently move around the hospital and use the nurse call system. However, if the portable nurse call buttons would be introduced in this department, the walking behavior of the patients and nurses might change as these buttons give the patients more freedom to walk around. The usage of the nurse call buttons might also change as patients would be able to make calls from anywhere in the hospital.

Embedding a new technology into practice is not straightforward and needs to be treated with care. The adoption rate of using Information and Communication Technology (ICT) to improve the quality of care is still very low [42,43]. One of the main reasons for this slow adoption rate is the gap in communication between the ICT and medical domain. These projects unite people with different backgrounds, such as software developers, health service researchers and nurses. Uniting all these people in a team requires effort and commitment to overcome the gap in communication. This problem can be approached by using bridge personnel who have the knowledge of multiple disciplines used in this process [44]. However, this personnel is often difficult to find.

To increase the adoption rate, the *oNCS system *can be introduced in several phases. Each phase should be supported with the needed training for the staff members and user research to adapt the system to the needs and feedback of the users.

In the first phase, the *oNCS system *software can be introduced in one department. In this phase, the new GUI is installed on the computer of the head nurse, which he/she can use to input the needed information about the staff members and patients, and the nurses are provided with PDAs to replace their portable phones or beepers. However, the mobile, portable nurse call buttons are not introduced yet to the patients. The patients keep using the nurse call buttons fixed to the walls of their room. Nevertheless, the novel nurse call algorithm is already deployed.

It is important to pick an appropriate department to test the new technology. Several criteria should be taken into account, such as openness to embrace new technology, the current usage of the nurse call system and the number of patients and nurses. It would be good to introduce the technology first in a department that would gain a lot of benefit from it. These are the departments in which there are few nurses compared to the number of patients and the patients make a reasonable amount of calls.

The most important consideration during this phase is the introduction of the GUI to the head nurse and the PDAs to the nurses. They should receive proper training to learn all the features of the GUI and the PDA. User research should also be conducted during this period, which explores the user-friendliness of the GUI and PDA. Both should be able to be customized to the preferences of the user and regular updates should be done taking the feedback of the nurses into account. It is important to emphasize to the head nurse the importance of entering all the data about the patients correctly such as their risk factors. However, a lot of the data about the patients can already be collected from the Electronic Health Record (EHR). Entering the data about the patient might seem like a tedious job for the head nurse as it introduces extra work. Therefore it is of vital importance to illustrate the benefits it introduces.

The nurses will also have to change their behavior towards receiving a call. Now they are used to often ignoring the call as multiple nurses receive it. They should be made aware that only one person receives the call at a time in the new system and that this nurse is the most appropriate person to handle the call at that time. They should only ignore it if they cannot leave their current task behind. After the time-out time another nurse will be called. This change might not be straightforward. It is important to illustrate the advantages of the new nurse call algorithm to improve adoption. This could be done by organizing sessions between the user researchers and the nurses in which several real-life examples are shown and both nurse call algorithms are discussed. As a result the nurse call algorithm could also be updated to better suit the needs of the nurses.

When the software system is properly adopted in the first department, the second phase can start. In this phase, the portable, mobile software buttons are introduced to the patients. The patients can now freely roam through the hospital and still make calls.

This is perhaps the most invasive change. It is important to convey to the patients not to abuse the system. When they are far away from the department, they should only make calls for urgent, medical calls and not for example for a glass of water. Otherwise nurses might have to walk long distances to answer simple calls, which might be rather frustrating.

Nurses can now also be called for patients who are not in their department e.g. because a patient becomes unwell inside a staircase far away from his/her own department. The implications of this should be thoroughly studied e.g. rules for responsibilities for patients.

In the third phase, the *oNCS system *can be gradually introduced into other departments of the hospital. The adoption rate in these other departments should be quicker, as the system has been thoroughly tested in the first department. Moreover, this department can be used as an illustration of the advantages of the system.

## Conclusion

This article showed that the current nurse call algorithms could be significantly improved by storing profile information about the staff members and patients in an ontology. Moreover, it introduces a software system that could easily be used to introduce portable nurse call buttons, which improve the mobility of patients, location-awareness and safety.

The person-oriented nature of the platform was clearly illustrated by using the context information about the risk factors of a patient to dynamically determine the priority of the call this patient is making. By using probabilistic reasoning algorithms, the probability that a specific call made by a specific patient has a specific priority can be determined. These probabilities are derived from the different risk factors of this patient as these risk factors will influence the probability that a patient makes urgent calls. All these probabilistic values are combined in an intelligent manner to determine the most suitable priority for this call.

The benefits of this novel *oNCS system *are illustrated with realistic simulations about data collected from the Ghent University Hospital. The *oNCS system *significantly improves the assignment of nurses to calls. Calls generally have a nurse present faster, the workload-distribution amongst the nurses improves and the priorities and kinds of the calls are taken into account. The execution time of the nurse call algorithm is negligible. However, before the system can be widely deployed, it is important that first a thorough study is done to characterize the correlation between the risk factors of patients and the reasons for their calls.

Future work will mainly focus on improving the scalability of the probabilistic assessment algorithm to determine the priority of a call. Simultaneously, hard-ware and algorithms for the effective and accurate determination of the location of staff members and patients will be further studied. Finally, the performance and benefits of the system will be thoroughly studied by performing realistic tests on the large-scale sensor network available within the IBCN research group.

## List of abbreviations used

A-BOX: Assertional Box; CASP: Context-Aware Service Platform; CI: Confidence Interval; COPD: Chronic Obstructive Pulmonary Disease; CT: Computed Tomography; DL: Description Logic; EPR: Electronic Patient Record; FOL: First-Order Logic; GB: GigaByte; GHz: GigaHertz; GUI: Graphical User Interface; IBCN: Information Technology Broadband Communication Networks; ICU: Intensive Care Unit; ID: IDentification; IV: IntraVenous; Max.: Maximum; MB: MegaByte; MEBN: Multi Entity Bayesian Network; MHz: MegaHertz; Min.: Minimum; ms: milliseconds; N3: Notation 3; Nr.: Number; oNCS: Ontology-based Nurse Call System; OSGi: Open Service Gateway initiative; OWL: Ontology Web Language; PC: Personal Computer; PDA: Personal Digital Assistant; Pr-OWL: Probabilistic OWL; RAM: Random-Access Memory; RDF: Resource Description Framework; RPM: Revolutions Per Minute; sec.: seconds; Serial ATA: Serial Advanced Technology Attachment; SPARQL: Sparql Protocol And RDF Query Language; Std. Err.: Standard Error; T-Box: Terminological Box; Turtle: Terse RDF Triple Language; W3C: World Wide Web Consortium; XML: eXtensible Markup Language.

## Competing interests

The authors declare that they have no competing interests.

## Authors' contributions

FO and DM carried out the study, participated in the development of the concepts described in this paper and drafted the manuscript. JD and PV participated in the case study. FDT, TD, TD and DVG supervised the study, participated in its design and coordination and helped to draft the manuscript. All authors read and approved the final manuscript.

## Pre-publication history

The pre-publication history for this paper can be accessed here:

http://www.biomedcentral.com/1472-6963/11/26/prepub
